# Cognitive Performance and Incident Alzheimer’s Dementia in Men Versus Women

**DOI:** 10.14283/jpad.2023.90

**Published:** 2024

**Authors:** I. Liampas, V. Siokas, C.G. Lyketsos, E. Dardiotis

**Affiliations:** 1. Department of Neurology, University Hospital of Larissa, School of Medicine, University of Thessaly, Larissa 41100, Greece;; 2. Department of Psychiatry and Behavioral Sciences, Johns Hopkins School of Medicine, Baltimore, MD 21205, USA;; 3. Richman Family Precision Medicine Center of Excellence in Alzheimer’s Disease, Johns Hopkins School of Medicine, Baltimore, MD 21205, USA.

**Keywords:** Sex differences, episodic memory, executive function, verbal fluency, naming

## Abstract

**BACKGROUND:**

The utility of neuropsychological measurements as forerunners of Alzheimer’s Disease Dementia (AD) in individuals with normal cognition or mild cognitive impairment (MCI) is undeniable.

**OBJECTIVES:**

To assess the differential prognostic value of cognitive performance in older men versus women.

**DESIGN:**

Longitudinal analysis of data acquired from the National Alzheimer’s Coordinating Center Uniform Data Set.

**SETTINGS:**

Data on older adults (≥60 years) were derived from 43 National Institute on Aging - funded Alzheimer’s Disease Research Centers.

**PARTICIPANTS:**

10,073 cognitively unimpaired (CU) older adults followed for 5.5±3.8 years and 3,925 participants with amnestic MCI monitored for 3.5±2.8 years.

**MEASUREMENTS:**

The domains of episodic memory, verbal fluency, naming, attention, processing speed and executive function were assessed. Cox proportional hazards models examined associations between individual cognitive domains and AD incidence separately for each participant set.

**CU and MCI.:**

These predictive models featured individual neuropsychological measures, sex, neuropsychological measure by sex interactions, as well as a number of crucial covariates.

**RESULTS:**

Episodic memory and verbal fluency were differentially related to future AD among CU individuals, explaining a larger proportion of risk variance in women compared to men. On the other hand, naming, attention and executive function were differentially related to future AD among participants with MCI, accounting for a greater fraction of risk variance in men than women.

**CONCLUSION:**

Cognitive performance is differentially related to risk of progressing to AD in men versus women without dementia.

## Introduction

Sex differences are increasingly recognised in multiple aspects of Alzheimer’s disease dementia (AD) (1). A constellation of genetic, hormonal and socio-cultural factors appears to shape the different risk (or resilience) of men or women for AD and lead to heterogeneous clinical courses (2–8). Unravelling the complex network of sex interactions in AD might provide the basis for the development of precision medicine approaches with individualized preventive and therapeutic interventions (9, 10).

Neuropsychiatric and cognitive impairments, the cardinal clinical manifestations of AD, differ significantly between men and women. The overall burden of behavioral and psychological disturbances is greater in female patients with AD (11). Psychotic and affective manifestations are more prevalent in women and apathy is more common among men (12). Affective disorders, lability symptoms and apathy are differentially associated with incident AD in cognitively unimpaired (CU) men versus women (13). Similarly, cognitive deficits are more conspicuous among women with AD. A small to moderately sized female disadvantage is consistently reported on global cognition as well as on most individual domains, involving verbal and non-verbal memory, visuospatial perception and language (14–16). Intriguingly the aforementioned differences in patients with AD appear to contradict the long-established female advantages in verbal memory and language among CU older individuals, while are in line with the well-established male advantage on visuospatial tasks (17–19).

The utility of specific neuropsychological measures as forerunners of AD in individuals with normal cognition or mild cognitive impairment (MCI) is clear (20–22). However, despite the aforementioned evidence on sex-specific neuropsychological patterns in the courses of normal ageing and AD, the predictive value of cognitive performance has not been explored separately in men and women. The present study aimed to assess the differential prognostic value of cognitive performance in male versus female older adults. We analysed the Uniform Data Set (UDS), a collaborative database instituted and monitored by the National Alzheimer’s Coordinating Center (NACC). The UDS is a central repository of longitudinally collected, standardised data from multiple National Institute on Aging/NIH - funded Alzheimer’s Disease Research Centers (ADRCs) across the United States.

## Methods

Study reporting adhered to the STROBE reporting recommendations (Strengthening the Reporting of Observational Studies in Epidemiology) (23). The rationale, design, data collection process as well as other key features of the UDS have been described elsewhere (24–26). In short, UDS operates since 2005 with a primary aim to promote and facilitate research in the cognitive disorders field. Clinician-, self- or family-referred volunteers, as well as actively recruited individuals with a cognitive status ranging from normal cognition to full-blown dementia are enrolled in accord with each ADRC’s discrete protocol and undergo standardized, comprehensive assessments on an approximately yearly basis. Written, informed consent is obtained from all participants or surrogates before participation. The Institutional Review Boards of each ADRC monitors all procedures in accordance with the ethical standards of the 1964 Declaration of Helsinki and its later amendments.

### Eligibility Criteria and Participant Selection

This analysis focused on data from older (≥60 years) NACC participants, enrolled between September 2005 (year UDS was established) and December 2021 (data freeze) from a total of 43 ADRCs. Participants with unimpaired cognition and amnestic MCI (significantly more likely to convert to AD compared to other subtypes of MCI) were separately analysed (27). The CU group included all individuals with normal cognition on NACC entry. The aMCI group involved those diagnosed with aMCI at entry to the NACC plus those with normal cognition at baseline who converted to aMCI at follow-up. For the latter group, the starting point of monitoring coincided with the first visit at which they were diagnosed with aMCI. Participants who went on to develop dementia other than AD at follow-up were excluded from the current study (due to the competing risks between AD and non-AD dementias).

The diagnoses of CU, MCI and dementia were established by either the examining physician or (in the vast majority) by an expert-consensus panel, in accord with the discrete protocol of each ADRC, using standard clinical criteria (28–33). Cognitively impaired participants who did not clearly fit into the categories of MCI or dementia were identified as cognitively impaired – not MCI. Participants were classified as CU in the absence of cognitive impairment (dementia, MCI or cognitive impairment not MCI). Individuals receiving FDA-approved medications for AD (i.e., tacrine, donepezil, rivastigmine, galantamine and memantine) were excluded from both participant sets (the prescription of such medication raised doubts about the credibility of the clinician-based diagnoses). Participants reporting a personal medical history of psychiatric disorders (schizophrenia, bipolar disorder, post-traumatic stress disorder, obsessive-compulsive disorder and developmental neuropsychiatric conditions) were also excluded from both groups to eliminate the confounding of long-standing neuropsychiatric manifestations that interfere significantly with cognition.

### Measurement of Cognitive Performance

All three UDS versions focused on the cognitive domains of episodic memory, verbal fluency, naming, attention, processing speed and executive function (26). In the first two versions of the UDS, episodic memory was assessed on the Logical Memory Test - Story A (LMT-SA) from the Wechsler Memory Scale—Revised (WMS-R) (34), naming according to the 30-item version of the Boston Naming Test (BNT-30) (35), verbal fluency on the total word production summing the Animal and Vegetable Fluency Tasks (36), attention using the Digit Span Test (DST, forward and backward conditions) from the WMS-R (34), processing speed on the Trail Making Test—Part A (TMT-A) and executive function on the Trail Making Test—Part B (TMT-B) (37). The administration and scoring of these tests has been detailed elsewhere (26). In the third most recent UDS version, verbal fluency, processing speed and executive function were evaluated on the same neuropsychological tasks, whereas episodic memory, naming and attention were assessed on the Craft Story 21 (CS-21) (38), Multilingual Naming Test (MINT) (39) and Number Span Test (NST, forward and backward conditions), respectively (40). The administration and scoring of these tests has been detailed elsewhere (40). To limit the amount of missing data, CS-21, MINT and NST scores were converted to LMT-SA, BNT-30 and DST scores correspondingly, according to the detailed conversion tables provided by the NACC crosswalk study (41).

### Factors and Covariates Considered

Chronological age at the beginning of the monitoring, years of formal education and body-mass index (BMI) were treated as scale variables. Sex, race (Caucasian, African American, American Indian or Alaska Native, Native Hawaiian or Pacific Islander, Asian and multiracial) and the following comorbidities, medications and daily habits were treated as categorical variables: history of traumatic brain injury (TBI), Parkinson’s disease (PD), seizures, cerebrovascular (CEVD) and cardiovascular disease (CAVD), atrial fibrillation (AF), hypertension, diabetes mellitus (DM), dyslipidaemia, smoking, alcohol or other substance abuse (with clinically significant impairment occurring over a 12-month period manifested in one of the following areas: work, driving, legal, or social) or vitamin B12 deficiency, and reported use of antidepressants, antipsychotics or anxiolytic/sedative/hypnotic agents (42, 43). These variables were evaluated based on subject or co-participant reporting (co-participants usually belonged to one of the following categories: spouse, partner, companion – child, sibling or other relative – friend, neighbour, colleague – paid caregiver) (44). To prevent over-adjustment, our analyses featured the variables of age, education, sex and race, along with those that qualified using a predefined statistical prerequisite (please see below, Statistical Analysis).

### Statistical Analysis

The present analysis focused on the following six neuropsychological measures: episodic memory [the number of items recalled in the delayed recall LMT-SA task (0–25 total items recalled)], naming [BNT-30 (0–30 items retrieved)], verbal fluency (the sum of word production in the Animal and Vegetable 1-min Fluency Tasks), attention [the sum of the longest sequences in DST forward (0–8 digits) and backward (0–7 digits) conditions], processing speed [total time in TMT-A (0–150 s)] and executive function [total time in TMT-B (0–300 s)].

The relationship between cognitive performance and incident AD was examined using adjusted Cox proportional hazards models. Participants who did not progress to dementia were censored at their last assessment. The proportionality of hazards assumption over time was explored for each model via Cox regression models with time dependent covariates. For example, the proportionality of hazards for episodic memory was tested using an extended Cox model featuring the term episodic memory*time along with episodic memory. The proportionality of hazards assumption was violated if the coefficient of the time interaction product was statistically significant.

Initially, two Cox proportional hazards models—one per participant group, CU and aMCI—were estimated for variable selection purposes. In specific, each model originally featured the aforementioned factors and covariates, and the least significant variables were consecutively eliminated in a backward manner, until all remaining variables in the model had a p<0.1. In this way, we aimed to balance between removing redundant variables (introducing a small drop in the proportion of variance captured) with including important ones (accounting for major confounders) while maintaining sufficient power for our analyses (preventing over-adjustment which would compromise precision).

Six independent analyses were conducted per participant group, one for each cognitive measure. In accord with the aforementioned statistical rule, all analyses involving CU participants featured the following factors and covariates: age, education, BMI, race, history of CEVD, TBI or seizures, reported use of antidepressants and sex, along with one neuropsychological measure at a time (e.g., episodic memory) including sex by neuropsychological measure interaction terms (e.g., sex*episodic memory). In the case of aMCI, all six survival analyses featured the following variables (that qualified from the variable selection process): age, education, BMI, race, history of PD, B12 deficiency or DM, reported use of antidepressants or anxiolytic/sedative/hypnotic agents and sex, along with one neuropsychological measure at a time (e.g., episodic memory) including sex by neuropsychological measure interaction terms (e.g., sex*episodic memory). Following the main analysis, a sensitivity analysis excluding the race categories with very low representation (American Indian or Alaska Native / Native Hawaiian or Pacific Islander) was performed. The results were practically identical and, therefore, only the findings of the main analyses were reported.

Baseline differences between those who did and those who did not progress to AD were analysed using independent sample t-tests (scale variables) and Pearson’s chi-squared tests (categorical variables). Results are provided separately for the CU and aMCI sets. Statistical analyses were performed using the IBM SPSS Statistics Software Version 26 (Chicago, IL, USA). The conventional threshold of α = .05 was implemented for the revelation of statistical significance.

## Results

### CU Participant Characteristics and Missing Data

The December 2021 data freeze of the UDS featured a total of 44,713 participants. Of them, 11,018 CU individuals at entry were eligible for the current analysis (Figure S1). Of these, 785 were excluded due to missing data on covariates; an additional 160 were excluded from all models because of missing data on all cognitive measures. Of the remaining 10,073, between 57 and 155 individuals were excluded from each survival analysis (due to missing data on the specifically analysed neuropsychological measurement). In the course of the 5.5±3.8 -year follow-up (range: 0.4 – 15.9 years), 665 older CU adults progressed to AD. Baseline differences between those who did and did not develop AD are in Table 1. The mean age of those who did not convert to dementia was 72.7±7.5 years (73.3±7.6 years for men and 72.4±7.5 years for women), whereas those who progressed to AD were on average 78.3±7.2 years (men were 78.0±6.7 years and women were 78.4±7.4 years).

CU individuals with missing data (n=945) were older (74.4±8.8 vs. 73.1±7.6 years), less educated (15.5±3.3 vs. 15.8±2.9 years) and performed worse on every cognitive task. Furthermore, they were more often of Caucasian and Asian ancestry (those without missing data were more frequently Black). CAVD, CEVD and DM were more prevalent among those with missing data while dyslipidaemia was less common (missing data analysis not shown).

### Cognitive Performance and Incident AD in CU Individuals by Sex

**Figure 1.**
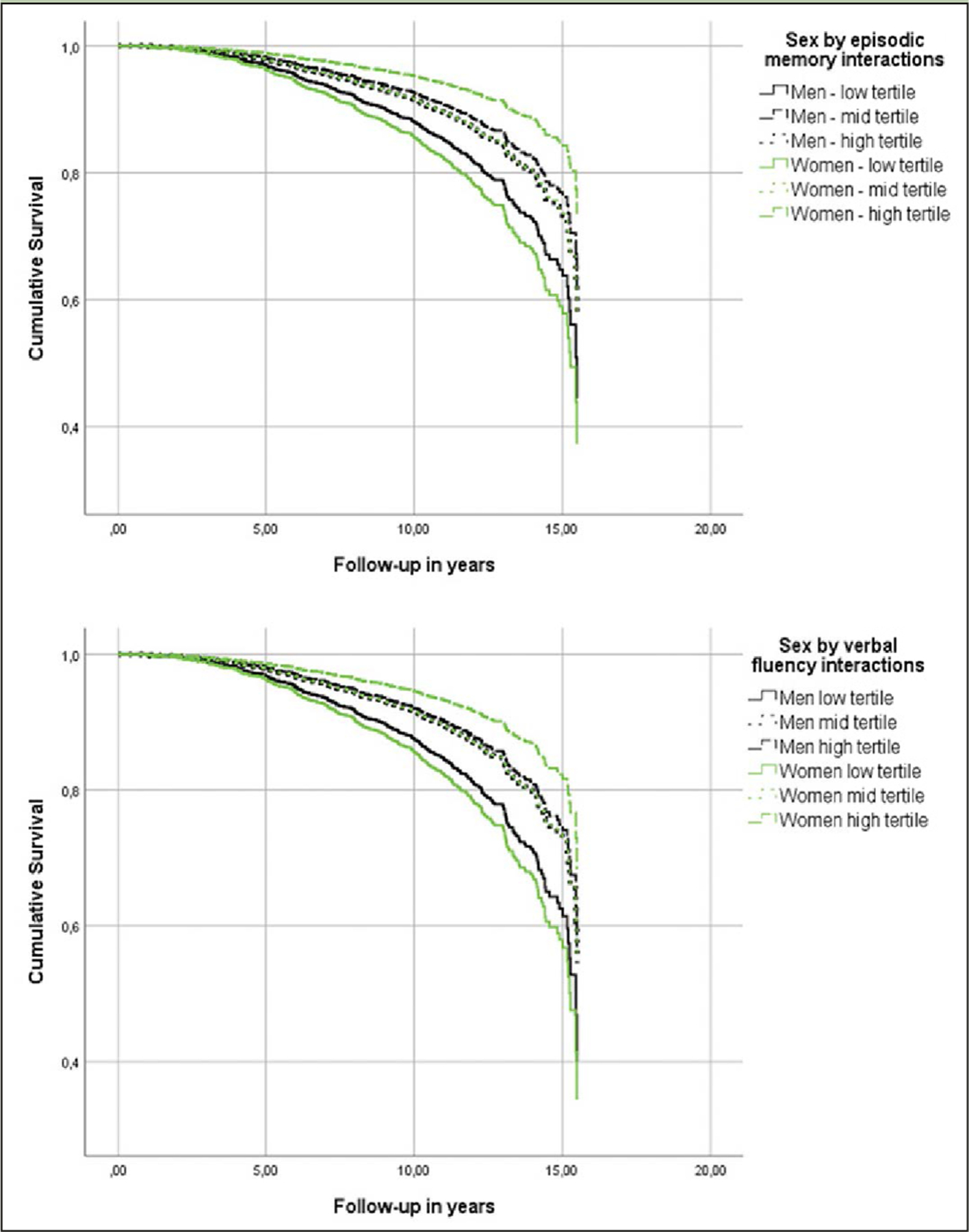
Heterogeneous associations of episodic memory and verbal fluency with incident Alzheimer’s disease dementia in cognitively unimpaired men and women Models were adjusted for age, education, BMI, race, history of CEVD, TBI or seizures and reported use of antidepressants.

While every neuropsychological measure was related to the hazard of AD (Table 2, main effects), only episodic memory and verbal fluency were differentially associated with the risk of incident AD in men versus women (Table 2, sex by neuropsychological measurement interactions & Figure 1). In specific, for each additional item recalled in the episodic memory task, the risk of future AD was reduced by 6% in women compared to men (Table 2). Regarding verbal fluency, each additional item generated in category fluency tasks reduced the hazard of progressing to AD by an additional 3% among women in comparison with men (Table 2). On the other hand, naming, attention, processing speed and executive function were comparably associated with future AD in both sexes (higher naming and attention scores reduced the hazard of AD conversion, while longer sessions on the processing speed and executive function tasks elevated the risk of developing AD at follow-up) (Table 2, main effects).

### MCI Participant Characteristics and Missing Data

Of the 44,713 participants in the UDS, 5,087 with aMCI were eligible for the current study (Figure S1). A total of 1,070 were not analysed because of missing data on confounders and an additional 151 due to missing data on all neuropsychological measurements. Among the remaining 3,925 participants, 33 to 179 individuals were excluded from each survival analysis (due to missing data on the specifically analysed cognitive measure). Throughout the average follow-up of 3.5±2.8 years (range: 0.4 – 15.5 years), 1,415 older adults with aMCI progressed to AD. Baseline differences between those who did and did not develop AD are in Table S1. The mean age of those who did not convert to dementia was 75.0±8.0 years (75.0±7.8 years for men and 74.9±8.1 years for women), whereas those who progressed to AD were on average 77.8±7.8 years (men were 77.0±7.2 years and women were 78.5±8.2 years).

Individuals with missing data (n = 1,162) were older (77.8±8.7 vs. 76.0±8.0 years) and performed better on episodic memory, while were more often women, of Asian ancestry compared to those without missing data. Dyslipidaemia, DM, alcohol or other substance abuse were more prevalent among those with missing data (missing data analysis not shown).

### Cognitive Performance and Incident AD in Individuals with aMCI

**Figure 2.**
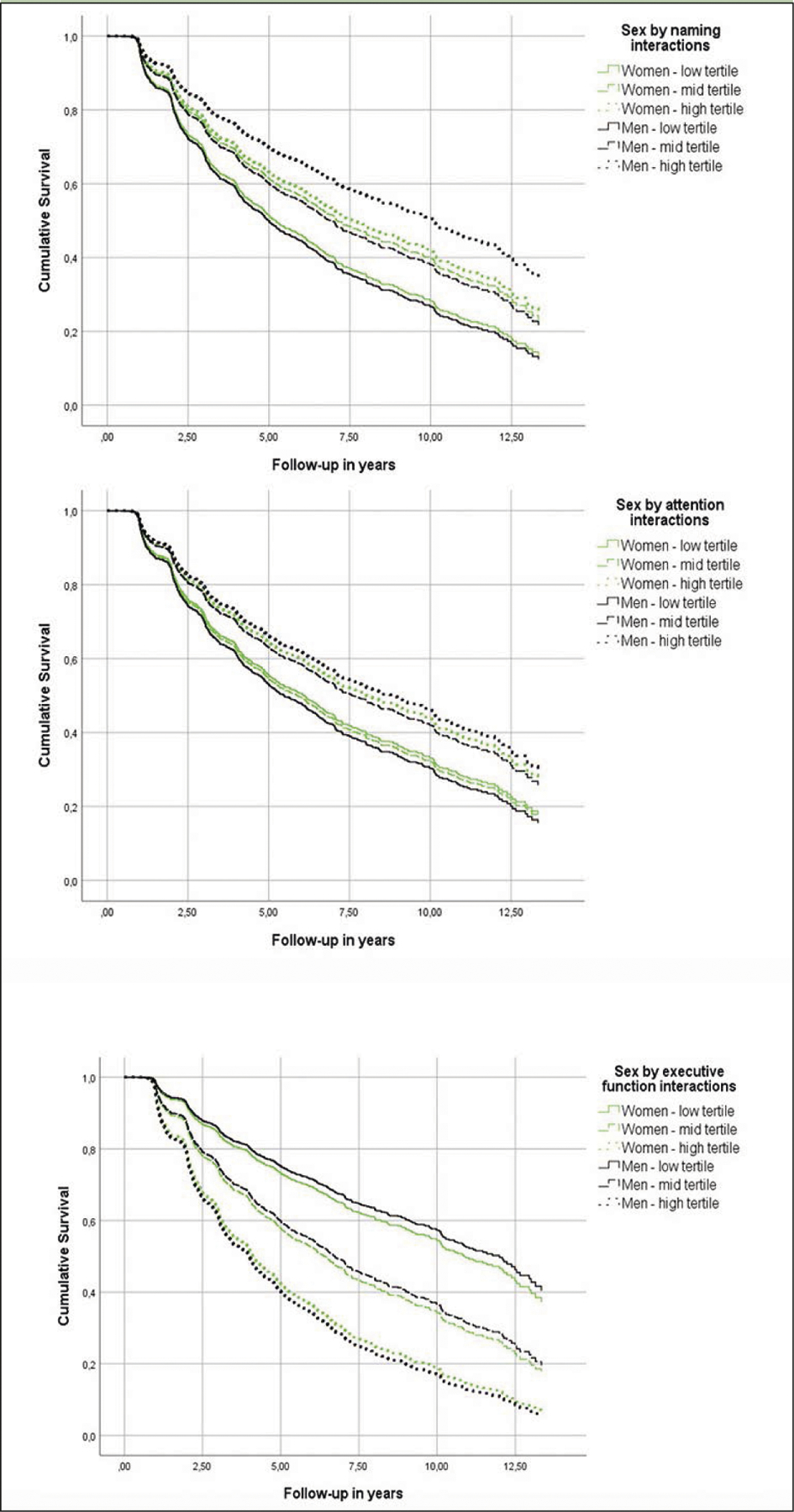
Heterogeneous associations of episodic memory and verbal fluency with incident Alzheimer’s disease dementia in cognitively unimpaired men and women Models were adjusted for age, education, BMI, race, history of PD, B12 deficiency or DM and reported use of antidepressants or anxiolytic/sedative/hypnotic agents.

Although all cognitive measures were associated with the risk of incident AD (Table 3, main effects), naming, attention and executive function were differentially related to the risk of AD in men versus women (Table 3, sex by neuropsychological measurement interactions & Figure 2). In specific, for each additional item recalled in the naming task, the risk of future AD was reduced by an additional 3% in men compared to women (Table 3). Moreover, each extra item scored in the attention task decreased the hazard of converting to AD by an additional 6% among men in comparison with women (Table 3). With respect to executive function, each additional second required in completing the TMT-B, increased the risk of future AD by an additional 1% in male compared to female participants (Table 3). On the other hand, episodic memory, verbal fluency and processing speed were similarly related to future AD in both sexes (higher episodic memory and verbal fluency scores reduced the hazard of developing AD at follow-up, while longer sessions on the processing speed task increased the risk of incident AD) (Table 3, main effects).

## Discussion

We report that neuropsychological measures are differentially related to the risk of progressing to AD in men versus women without dementia. In CU older individuals, episodic memory and verbal fluency performance explained a larger proportion of the risk variance in women compared to men; in other words, each additional item scored in both tasks mitigated the hazard of future AD more among women than men. On the other hand, naming, attention and executive function performances accounted for a greater fraction of the risk variance in men compared to women with aMCI, i.e., each extra point in the naming and attention totals reduced the hazard of future AD more among men than women; and, each additional second required to complete the executive function test increased the risk of incident AD more prominently among men than women.

The identification of biological and clinical markers optimized for early detection of individuals at high-risk of AD has preoccupied research for many years. Although biomarkers (genetic, cerebrospinal fluid, imaging) are excellent predictors of AD, their costs or interventional nature tempers widespread use (45). It is worth noting that novel blood-based biomarkers of AD are more cost efficient and less invasive, offering larger-scale applicability, easier repeat screening and monitoring, as well as increased patient acceptance (46). Among them, amyloid-β, tau, phospho-tau, GFAP (glial fibrillary acidic protein) and Nf-L (neurofilament light) are the most promising ones (47, 48). However, there are still several remaining challenges impeding the widespread use of the aforementioned markers such as the lower concentrations of CSF based markers in plasma, which necessitates the use of detection methods with substantially higher analytical sensitivity.

On the other hand, clinical assessments offer a readily available cost-effective, easily applicable, non-invasive screening alternative. The core clinical manifestations of AD, cognitive decline and/or neuropsychiatric symptoms, tend to precede the onset of dementia by one to two decades (49, 50). While these clinical markers may fall short in terms of predictive potential compared to biological markers, they apparently outcompete laboratory indices in applicability and patient acceptance.

The utility of neuropsychological markers in the prodromal detection of high-risk individuals is well-established. Published evidence suggests consecutive compromise of episodic memory, language and visuospatial skills, followed by attention, processing speed and executive function, in the pre-dementia course of the disease continuum (20, 51–53). Accordingly, episodic memory and (secondarily) verbal fluency performance are among the strongest predictors of later AD in individuals with normal cognition and aMCI (54, 55). The remaining neuropsychological measures do not seem to enhance the overall predictive sensitivity considerably; however, it is argued that they add to the predictive specificity and positive prognostic value of episodic memory and verbal fluency assessments (20, 55).

We aimed to enhance the prognostic properties of preclinical neuropsychological evaluations via the exploration of sex-specific patterns. The identification of cognitive measures that better capture risk variances of incident AD in men versus women before dementia, could theoretically lead to superior risk stratification approaches. Combining neuropsychological with additional clinical (most notably neuropsychiatric symptoms) or biological markers of well-established prognostic quality, might lead to even more accurate predictive models (13). Future research ought to confirm and combine these findings in the search of a more sensitive and precise predictive model that will serve preclinical detection purposes of individuals without dementia at high-risk of converting to AD.

### Strengths and Limitations

The size of our sample, the long follow-up period and the large number of incident AD cases are the study’s main strengths. We were careful to exclude individuals under treatment with FDA-approved medication for AD as well as those with psychiatric disorders that may interfere with cognition. Moreover, our analytical approach accounted for numerous important confounders.

It is also appropriate to point limitations. First, the diagnostic procedures did not involve the use of any biomarkers; therefore, misclassification bias may exist in the diagnostic categorization of dementias. Second, although the analytic approach accounted for numerous important factors and covariates, our findings may have been still driven by residual confounding (56). Of note, different sets of covariates were used in the analysis of the CU and MCI participants sets, limiting the comparability of the results from the two analyses. Also, a sizeable fraction of the NACC participants was excluded due to missing data. An additional weakness is the observational design; irrespective of the temporal association of our findings, it is not possible to make etiologic inferences, especially when considering that neurodegenerative alterations in the brain tend to precede the formal identification of AD for many years (20). Finally, the focus of the current report was arguably limited; both in terms of exposures and outcomes. Therefore, future research ought to investigate sex differences with respect to the prognostic value of visuospatial ability on incident AD (the first two versions of the UDS did not comprehensively collect data on visuo-perceptual skills), as well as explore other dementia entities, apart from AD.

## Conclusions

We report that episodic memory and verbal fluency are differentially associated with risk of conversion to AD in CU men versus women. On the other hand, we demonstrated that naming, attention and executive function are differentially related to hazard of future AD in men versus women with aMCI. Our findings have implications for the early identification of individuals with normal cognition and aMCI at high risk of incident AD.

## Supplementary Material

1920866_SUP 1

1920866_SUP 2

## Figures and Tables

**Table 1.** Baseline differences between cognitively unimpaired individuals who did and did not develop Alzheimer’s disease dementia (AD) at follow-up

Variable	Without dementia at follow-up (n=9,408)	AD at follow-up (n=665)	P-value
Age in years	72.7 ±7.5	78.3 ±7.2	< .001
Formal education in years	15.9 ±2.9	15.3 ±2.9	< .001
Body-mass index	27.4 ±5.3	26.3 ±4.9	< .001
Sex (female/male)	65.8 / 34.2 %	66.9 / 33.1 %	.555
Race (Caucasian / African American / American Indian or Alaska Native / Native Hawaiian or Pacific Islander / Asian / Multiracial)	79.1 / 15.1 / 0.4 / 0.1 / 2.5 / 2.9 %	86.2 / 10.2 / 0.0 / 0.0 / 1.1 / 2.6 %	< .001
Parkinson’s disease	1.6 %	0.5 %	.016
Traumatic brain injury	10.0 %	9.3 %	.572
History of seizures	1.4 %	2.3 %	.072
B12 deficiency	4.4 %	4.4 %	.986
Alcohol abuse	2.7 %	2.1 %	.346
Other substance abuse	0.8 %	0.2 %	.071
Smoking history	45.1 %	44.2 %	.686
Cardiovascular disease	10.2 %	13.5 %	.007
Cerebrovascular disease	5.3 %	10.1 %	< .001
Atrial Fibrillation	6.3 %	7.3 %	.328
Diabetes Mellitus	12.4 %	10.7 %	.206
Hypertension	50.0 %	54.8 %	.016
Dyslipidaemia	55.2 %	51.3 %	.055
Intake of Antidepressants	16.4 %	17.6 %	.414
Intake of Antipsychotics	0.4 %	0.6 %	.415
Intake of Anxiolytics/sedatives/hypnotics	10.7 %	8.4 %	.063
Episodic memory (items)	12.6 ±4.1	10.6 ±4.4	< .001
Naming (items)	27.3 ±3.1	26.2 ±3.8	< .001
Verbal fluency (words)	35.3 ±8.4	31.3 ±8.1	< .001
Attention (digits)	11.6 ±2.0	11.3 ±2.0	< .001
Processing speed (seconds)	34.3 ±14.8	39.7 ±17.8	< .001
Executive function (seconds)	89.4 ±47.2	109.4 ±58.7	< .001

**Table 2.** Associations between neuropsychological measurements and incident Alzheimer’s disease dementia in cognitively unimpaired individuals

Variable	Hazard ratio	Lower 95%CI	Upper 95%CI	p-value
Sex	2.03	1.29	3.20	.002
Episodic memory	0.92	0.89	0.95	< .001
Sex by episodic memory	0.94	0.91	0.98	.004
Sex	1.62	0.55	4.75	.378
Naming	0.94	0.91	0.98	< .001
Sex by naming	0.98	0.94	1.02	.252
Sex	1.75	1.43	2.16	.008
Verbal fluency	0.96	0.95	0.98	< .001
Sex by verbal fluency	0.97	0.95	0.99	.010
Sex	0.91	0.36	2.30	.837
Attention	0.93	0.86	0.99	.023
Sex by attention	1.00	0.92	1.09	.988
Sex	0.79	0.53	1.16	.230
Processing speed	1.010	1.002	1.019	.016
Sex by processing speed	1.004	0.994	1.013	.437
Sex	0.69	0.49	0.98	.037
Executive function	1.003	1.001	1.006	.020
Sex by executive function	1.003	1.000	1.006	.081

Models were adjusted for age, education, BMI, race, history of CEVD, TBI or seizures and reported use of antidepressants. First the main effects of sex (male sex was used as the reference category) and neuropsychological measurements are provided and then sex by neuropsychological measurements interactions are quoted (male sex by neuropsychological measurements interactions was used as the reference category).

**Table 3.** Associations between neuropsychological measurements and incident Alzheimer’s disease dementia in individuals with amnestic mild cognitive impairment

Variable	Hazard ratio	Lower 95%CI	Upper 95%CI	p-value
Sex	0.93	0.77	1.52	.424
Episodic memory	0.88	0.86	0.89	< .001
Sex by episodic memory	0.99	0.96	1.01	.343
Sex	2.22	1.23	3.98	.008
Naming	0.95	0.93	0.96	< .001
Sex by naming	0.97	0.95	0.99	.007
Sex	0.89	0.59	1.35	.587
Verbal fluency	0.94	0.93	0.95	< .001
Sex by verbal fluency	1.00	0.98	1.01	.851
Sex	1.82	1.01	3.29	.048
Attention	0.95	0.92	0.99	.011
Sex by attention	0.94	0.89	0.99	.027
Sex	0.80	0.63	0.99	.047
Processing speed	1.010	1.007	1.013	< .001
Sex by processing speed	1.004	0.999	1.008	.113
Sex	0.80	0.64	1.00	.052
Executive function	1.005	1.004	1.006	< .001
Sex by executive function	1.001	1.000	1.003	.048

Models were adjusted for age, education, BMI, race, history of PD, B12 deficiency or DM and reported use of antidepressants or anxiolytic/sedative/hypnotic agents. First the main effects of sex (female sex was used as the reference category) and neuropsychological measurements are provided and then sex by neuropsychological measurements interactions are quoted (female sex by neuropsychological measurements interactions was used as the reference category).

## Data Availability

For further information on access to the NACC database, please contact NACC (https://naccdata.org/).
