# Supplementary material for: Cognitive Performance and Incident Alzheimer’s Dementia in Men Versus Women
Source: J Prev Alzheimers Dis. 2023 Jul 13;11(1):162–70. doi: 10.14283/jpad.2023.90 (PMC10794850; doi:10.14283/jpad.2023.90)
Supplement: Supplementary file 1 — Table S1 Baseline differences between individuals with amnestic mild cognitive impairment who did and did not develop Alzheimer's disease dementia (AD) at follow-up. [file 42414_2023_258_MOESM1_ESM.docx]

**Table S1** Baseline differences between individuals with amnestic mild cognitive impairment who did and did not develop Alzheimer’s disease dementia (AD) at follow-up.

| **Variable** | **Without dementia at follow-up (n=2,510)** | **AD at follow-up**  **(n=1,415)** | **P-value** |
| --- | --- | --- | --- |
| Age in years | 75.0 ±8.0 | 77.8 ±7.8 | **< .001** |
| Formal education in years | 15.4 ±3.3 | 15.4 ±3.2 | .915 |
| Body-mass index | 27.3 ±5.0 | 25.9 ±4.5 | **< .001** |
| Sex (male/female) | 48.9 / 51.1 % | 45.8 / 54.2 % | .063 |
| Race (Caucasian / African American / American Indian or Alaska Native / Native Hawaiian or Pacific Islander / Asian / Multiracial) | 77.6 / 16.1 /  0.4 / 0.0 /  2.5 / 3.3 % | 84.7 / 10.3 /  0.1 / 0.1 /  2.3 / 2.5 % | **< .001** |
| Parkinson’s disease | 2.6 % | 0.6 % | **< .001** |
| Traumatic brain injury | 13.7 % | 11.0 % | **.016** |
| History of seizures | 2.4 % | 1.9 % | .285 |
| B12 deficiency | 5.7 % | 6.1 % | .563 |
| Alcohol abuse | 4.2 % | 3.2 % | .126 |
| Other substance abuse | 1.1 % | 0.4 % | **.025** |
| Smoking history | 46.8 % | 46.6 % | .442 |
| Cardiovascular disease | 16.0 % | 14.1 % | .124 |
| Cerebrovascular disease | 10.6 % | 10.0 % | .573 |
| Atrial Fibrillation | 9.4 % | 8.2 % | .210 |
| Diabetes Mellitus | 16.5 % | 13.9 % | **.030** |
| Hypertension | 56.9 % | 57.6 % | .654 |
| Dyslipidaemia | 62.6 % | 60.1 % | .127 |
| Intake of Antidepressants | 22.7 % | 21.4 % | .334 |
| Intake of Antipsychotics | 1.2 % | 1.2 % | .927 |
| Intake of Anxiolytics/sedatives/hypnotics | 12.6 % | 8.1 % | **< .001** |
| Episodic memory | 8.4 ±4.3 | 5.8 ±4.5 | **< .001** |
| Naming | 25.6 ±4.1 | 24.6 ±4.7 | **< .001** |
| Verbal fluency | 28.8 ±7.5 | 26.0 ±7.3 | **< .001** |
| Attention | 10.9 ±1.9 | 10.8 ±1.9 | .081 |
| Processing speed | 40.8 ±19.9 | 45.3 ±22.3 | **< .001** |
| Executive function | 122.9 ±70.1 | 146.7 ±76.8 | **< .001** |
