# Supplementary material for: Cognitive Performance and Incident Alzheimer’s Dementia in Men Versus Women
Source: J Prev Alzheimers Dis. 2023 Jul 13;11(1):162–70. doi: 10.14283/jpad.2023.90 (PMC10794850; doi:10.14283/jpad.2023.90)
Supplement: Supplementary file 2 — Supplementary material, approximately 57.2 KB. [file 42414_2023_258_MOESM2_ESM.docx]

**Figure S1** Flowcharts of participant selection.

**2,349 men with amnestic** **MCI**

**2,738 women with amnestic** **MCI**

**5,087** eligible participants with amnestic MCI

**581** younger than 60 when first diagnosed with MCI, **1945** with psychiatric disorders or FDA approved meds for AD

**1,282** had non-amnestic MCI subtypes

**7,580** adults with MCI 60 years or older, without a physician-based diagnosis of psychiatric disorder or FDA-approved medication for AD

**490** progressed to other dementia entities, **721** had an inconclusive-missing dementia diagnosis

**6,369** participants with MCI

**7,239 CU women**

**3,779 CU men**

**11,018** eligible CU participants

**178** participants progressed to other dementia entities

**11,196** adults 60 years or older, without a physician-based diagnosis of psychiatric disorder or FDA-approved medication for AD

**13,723** CU individuals at baseline with at least one follow-up assessment

**1,570** participants younger than 60, **957** with psychiatric disorders or FDA approved meds for AD

**27,108** cognitively impaired participants at baseline, **3,882** participants without follow-up

**13,409** without follow-up, **9,329** with dementia at baseline

**10,106** participants with MCI at baseline or follow-up (without dementia prior to their first MCI diagnosis)

**11,869** without MCI at baseline or follow-up

**21,975** participants without dementia and at least one follow-up assessment

**44,713** total participants

**44,713** total participants
